# Effect of Donor and Recipient ABH-Secretor Status on ABO-Incompatible Living Donor Kidney Transplantation

**DOI:** 10.3389/fimmu.2021.671185

**Published:** 2021-06-14

**Authors:** Fan Zhang, Saifu Yin, Yu Fan, Turun Song, Zhongli Huang, Jiayu Liang, Jiapei Wu, Youmin Yang, Tao Lin, Xianding Wang

**Affiliations:** ^1^ Department of Urology/Institute of Urology, West China Hospital, Sichuan University, Chengdu, China; ^2^ Organ Transplantation Center, West China Hospital, Sichuan University, Chengdu, China

**Keywords:** kidney transplantation, ABO blood-group system, blood group incompatibility, graft function, accommodate

## Abstract

**Introduction:**

ABO blood group antigens within grafts are continuously exposed to anti-A/B antibodies in the serum of recipients after ABO-incompatible (ABOi) kidney transplantation and are instrumental in antibody-mediated rejection. Some individuals secrete soluble blood group antigens into body fluids. In this study, we investigated the effect of donor and recipient secretor status on the outcomes of ABOi kidney transplantation.

**Methods:**

Data of a total of 32 patients with ABOi living donor kidney transplantation were retrospectively collected between 2014 and 2020 in West China Hospital. The genotype and phenotype of both donors and recipients were examined and evaluated with post-transplantation anti-A/B titer changes, graft function, and rejection.

**Results:**

Of the 32 recipients and 32 donors, 23 (71.9%) recipients and 27 (84.4%) donors had secretor genotypes, whereas 9 (28.1%) recipients and 5 (15.6%) donors did not. Anti-A/B titers after ABOi kidney transplantation were not significantly influenced by the secretor status of either donors or recipients. The post-transplantation serum creatinine (Scr) levels and estimated glomerular filtration rate (eGFR) was better in weak- or non-secretor recipients at day 30 (Scr P = 0.047, eGFR P = 0.008), day 90 (Scr P = 0.010, eGFR P = 0.005), and month 9 (eGFR P = 0.008), and recipients from secretor donors had a lower incidence of graft rejection in the first year after ABOi transplantation (P = 0.004).

**Conclusions:**

A weak secretor status phenotype was found in both genotypes, i.e., individuals who secreted soluble antigens as well as those who did not. The recipient ABH-secretor status may have an influence on early posttransplant renal function, and the donor ABH-secretor status might affect the incidence of graft rejection.

## Introduction

Kidney transplantation from living donors has solved the problem of organ shortage to some extent (1, 2). However, approximately one third of potential living donors and recipients are ABO-incompatible (ABOi) (3). ABOi kidney transplantation is an alternative for cases when there are no ABO-compatible (ABOc) donors available. The initial development of ABOi kidney transplantation was hindered by the high rates of antibody-mediated rejection (AMR) after transplantation (4). Currently, ABOi is no longer considered a contraindication to kidney transplantation because of preconditioning protocols for the removal of anti-donor ABO antibodies (5). Long-term results show the same survival rate and organ function between ABOi and ABOc kidney transplantation (6).

To prevent AMR, pre-existing anti-A/B titers of recipients must be reduced to a safe range (e.g., ≤1:16 in most transplant centers) before ABOi transplantation (7–9). Two weeks after ABOi kidney transplantation, despite the presence of A/B antigen on the graft and the existence of corresponding antibodies in the recipient’s blood, there was no ABO antigen-antibody reaction and the ABOi allograft functioned normally. This tolerance is known as accommodation (10). However, when the anti-A/B titers were in a relatively safe range on the transplant day, some grafts still failed, while others survived the ABOi transplantation; therefore, the underlying mechanism needs to be elucidated (11).

In ABOi kidney transplantation, antigens A and B are mainly present in the vascular endothelium, distal convoluted tubules, and collecting ducts of the donor kidney. The β-galactoside α-1,2-fucosyltransferase, encoded by the *FUT2* gene, is an enzyme required for the final step in the soluble A and B antigen synthesis pathway (12, 13). Individuals with *FUT2* are capable of secreting soluble ABH blood group antigens into body fluids, including saliva (the most abundant), urine, tears, gastric juice, bile, amniotic fluid, serum, semen, sweat, and breast milk (14). For recipients whose blood types are blood group O, H antigens are secreted into body fluid instead of A or B antigens, which are detectable in the body fluid of individuals with blood types A and B, respectively. After ABOi kidney transplantation, renal grafts can secrete soluble A/B/H antigens into the blood of recipients according to the donor’s secretor status (15). However, the influence of the secretion status of donors/recipients on recipient accommodation remains controversial (16, 17). In this study, we investigated the correlation between the secretion status of donors/recipients and the prognosis of ABOi transplantation.

## Methods

### Study Population

Patients who underwent ABOi kidney transplantation between September 2014 and August 2020 at our institution voluntarily participated in this study. Both donors and recipients were tested for genotypes and phenotypes of the secretor status. We retrospectively collected and analyzed the clinical data of the included donor-recipient pairs. Each kidney transplantation procedure was approved by the institutional review board of West China Hospital and the Health Commission of Sichuan Province (18). Recipients with pretransplant donor-specific anti-HLA antibodies (DSA) were excluded from the study. The study protocol was approved by the local ethics committee (No.2019SHEN418).

### Measure for Genotype and Phenotype of Secretor Status

Peripheral blood was drawn from each donor and recipient pair before the kidney transplantation. The serum was then separated and used in laboratory tests to evaluate the secretor status. The genotypes of the donors/recipients were determined using PCR-based direct sequencing. The second exon of *FUT2*, which encodes the protein, was amplified by PCR using the amplification primers FUT2-F (5′-AGCGCCCCGGGCCTCCATCTCC-3′) and FUT2-R (5′-GGAACCATGTGCTTCTCATGCCCG-3′). The final volume of the PCR mixture was 20 μl, which contained 10 μl of GoTaqGreenMasterMix (Promega, USA), 6.8 μl of nuclease-free water, and 2 μl of sample DNA (approximately 50–100 ng). The final concentration of primers was 0.3 μmol/L. The reaction mixture was subjected to an initial denaturation at 94°C for 5 min, followed by 32 cycles of amplification (94°C for 30 s, 59°C for 30 s, 72°C for 90 s). The *FUT2* genotypes were then determined by two-way sequencing reaction on an ABI 3130 gene sequencer with a BigDye terminator v3.1 sequencing Kit (ABI, USA). The sequencing primers used were FUT2-F and FUT2-R, and the ethanol/sodium acetate method was used to purify the amplified products of the sequencing reaction.

The phenotype secretor status was determined using the Wiener agglutination test (19). A total of 5 to 10 ml of saliva was collected from the donor/recipient and stored in a sterile test tube. Afterward, the saliva was placed in a boiling water bath for 10 min and then extracted in a centrifuge at 2500 rpm for 10 min. The following samples were added into four different tubes: one drop of saliva and one drop of anti-A serum were added into the first tube, one drop of saliva and one drop of anti-B serum were added into the second tube, one drop of normal saline and one drop of anti-A serum were added into the third tube, and one drop of normal saline and one drop of anti-B serum were added to the fourth tube. After 20 min at room temperature (16–20°C), a drop of 5% suspension A erythrocytes was added to the first and third tubes, and a drop of 5% suspension B erythrocytes was added to the second and fourth tubes. After incubation for 20 min at room temperature (16–20°C), all tubes were examined for the presence or absence of hemagglutination. The absence of agglutination in the first and second tubes indicated the secretor phenotype. The same agglutination strength between the first two tubes and the last two tubes signifies the non-secretor phenotype, whereas a distinctly weaker agglutination in the first two tubes is regarded as the weak secretor phenotype.

### Immunosuppression and Preconditioning Principles

Triple oral immunosuppressive therapy including tacrolimus (Tac; 3 mg/day), mycophenolate mofetil (MMF; 1500 mg/day) or enteric-coated mycophenolate sodium (EC-MPS; 1080 mg/day), and prednisone (Pred; 5 mg/day) started 2 to 4 weeks before ABOi transplantation. According to the perceived immunologic risk, which depends on panel reactive antibodies (PRA), basiliximab (20 mg on days 0 and 4) or antithymocyte globulin (ATG; 1 mg/kg on days 0 to 3 or 0 to 4) were used. Preconditioning protocols used for ABOi living donor kidney transplantation were individualized according to the initial level of blood group antibody. Recipients whose initial blood group antibody (IgG and IgM) titers were less than 1:8 were pretreated with immunosuppressive agents alone. Recipients whose initial blood group antibody titer was equal to 1:16 received oral immunosuppressants and underwent plasma exchange/double filtration plasmapheresis (PE/DFPP). Recipients with initial blood group antibody titers ≥ 1:32 received oral immunosuppressants, intravenous rituximab, and PE/DFPP to ensure that the ABOi titer on the operation day was ≤ 1:8. Oral Tac and Pred were stopped on the transplant day, and the dose of MMF was increased to 2000 mg/day (MMF) or 1440 mg/day (EC-MPS). Intravenous methylprednisolone was administered intraoperatively at a dose of 500 mg, and at 200 mg/day on days 1 to 3 after transplantation, followed by oral Pred (60 mg/day, tapered to 5 mg/day within 2 weeks). Tac was re-initiated on post-transplant day 2. The target trough level of Tac was 5 to 10 ng/ml for the first 3 months, 4 to 8 ng/ml for months 4 to 12, and 4 to 6 ng/ml thereafter.

### Definition of Clinical Parameters

The initial anti-A/B antibody titer levels were defined as the anti-A/B antibody titer of the recipients prior to any immunomodulatory treatment. The pretransplant anti-A/B antibody titer was defined as anti-A/B antibody titer levels immediately prior to kidney transplantation. Posttransplant titers were monitored on days 1, 3, 7, 14, and also in months 1, 3, 6, 9, 12, 18, and 24 after transplantation. We used a gel card technique throughout the study period to measure the anti-donor IgM and IgG titers.

To explore the fluctuation of blood group antibodies after allogeneic ABOi kidney transplantation, we focused on the continuous level and the first change of anti-A/B antibody titers. The definition of titer elevation was the first time that one or more log2 serum titers of blood group antibody levels were promoted after transplantation. Similarly, titer reduction was defined as the first time one or more log2 serum titer reductions of the blood group antibody level. The time from the day of transplantation to titer elevation/reduction was also determined.

Renal function was measured by serum creatinine (Scr) levels and estimated glomerular filtration rate (eGFR), according to the CKD-EPI formula (20), which was measured on the transplantation day and on days 1, 3, 7, 14; and also in months 1, 3, 6, 9, 12, 18, and 24 after transplantation. Pretransplant Scr and pretransplant eGFR were defined as the Scr and eGFR levels on the transplantation day. Graft rejection was defined as a clinical diagnosis of graft rejection by clinical symptoms, such as oliguria or edema, or a significant increase in Scr of more than 50% within 3 days, which was not explained by other reasons. Biopsy was then performed in all the patients clinically diagnosed with graft rejection.

### Grouping Methods

Due to the variety of results with genotypes and phenotypes of secretor status, the posttransplant recipient anti-A/B titers and renal function were compared based on the following grouping methods marked I–IV: (a) I, recipients from genotype secretors *vs*. recipients from genotype non-secretors; (b) II, recipients from phenotype secretors *vs*. recipients from phenotype weak- or non-secretors; (c) III, genotype recipients *vs*. genotype non-secretor recipients; and (d) IV, phenotype recipients *vs*. phenotype weak- or non-secretor recipients. Since we found that the two cohorts divided by method III were exactly the same as those divided by method IV, the results between the two methods were also the same.

### Statistical Analysis

All analyses were performed using R software (version 3.4.4). The research results are presented as the mean ± standard deviation (SD) or median (range). Mean values of the groups of normally distributed data were compared using Student’s t-tests, whereas Wilcoxon rank-sum tests were used to compare non-normally distributed data. Furthermore, chi-square tests or Fisher’s exact tests were used to compare categorical variables. Survival curves were calculated by Kaplan–Meier analysis, and comparisons between groups were made using the log-rank test. Statistical significance was set at P < 0.05.

## Results

Of 98 consecutive patients who underwent living donor ABOi kidney transplantation at West China Hospital, Sichuan University, China, from September 2014 to August 2020, 32 patients and their corresponding donors participated in this study and underwent tests for both genotypes and phenotypes of secretor status before kidney transplantation. The distribution of the secretor genotypes or phenotypes is summarized in **Table 1**. Of the 32 recipients and 32 donors who met the inclusion criteria, 23 (71.9%) recipients were secretor genotypes and 9 (28.1%) were not, whereas 27 (84.4%) donors were secretor genotypes and 5 (15.6%) were not. All the nine non-secretor genotype recipients and five non-secretor genotype donors showed a weak-secretor or non-secretor phenotype, while 49/50 secretor genotype individuals presented a secretor phenotype (**Figure 3**). However, one donor with the secretor genotype presented a weak-secretor phenotype.

**Table 1:** The genotypes and phenotypes of secretor status in 32 recipients and 32 donors.

Features	N of donors (%)	N of recipients (%)
Genotypes		
Secretor	27 (84.4%)	23 (71.9%)
Se357/Se357,385	12 (37.5%)	11 (34.4%)
Se/Se357	5 (15.6%)	6 (18.8%)
Se357/Se357	4 (12.5%)	2 (6.3%)
Se/Se357,385	4 (12.5%)	3 (9.4%)
Se/Se	1 (3.1%)	0
Se95,357/Se357,385	1 (3.1%)	0
Se357/Se178,357,385	0	1 (3.1%)
Nonsecretor	5 (15.6%)	9 (28.1%)
Se357,385/Se357,385	5 (15.6%)	9 (28.1%)
Phenotypes		
Positive	26 (81.3%)	23 (71.9%)
Weak	4 (12.5%)	7 (21.9%)
Negative	2 (6.3%)	2 (6.3%)

The genotypes were determined by PCR-based direct sequencing, and Se357,385/Se357,385 was regarded as genotype non-secretor while other genotypes were regarded as secretors. The phenotypes were determined by the Wiener agglutination test.

The clinical characteristics of the 64 individuals (32 recipients and 32 donors) are presented in **Table 2**. The recipients were predominantly male (n = 23, 71.9%), and the donors were mainly female (n=26, 81.3%). The median age was 50 (32–67) for donors and 30.5 (20–43) for recipients. 23 (71.9%) underwent plasma transfusion. The median follow-up time in this study was 331.5 (range, 53–811) days. None of the 32 patients had undergone transplantation before. Furthermore, 5 of 32 patients had positive pretransplant PRA and received ATG therapy.

**Table 2:** Clinical characteristics of the study population.

Characteristics	All (n = 32)
Donor	
Age (median, range)	50 (32–67)
Male/female	6/26
BMI (mean, SD)	24.35 (3.34)
Blood group, n (%)	
A	13 (40.6%)
B	11 (34.4%)
AB	8 (25%)
O	0
Recipient	
Age (median, range)	30.5 (20–43)
Male/female	23/9
BMI, mean (SD)	21.07 (2.67)
Blood group, n (%)	
A	6 (18.8%)
B	8 (25%)
AB	0
O	18 (56.3%)
pre-transplant PRA	
Positive	5 (15.6%)
Negative	27 (84.4%)
Length of time on dialysis (mean SD)	22.63 (22.58) months
Initial IgG titer (median, range)	24 (0–512)
Initial IgM titer (median, range)	64 (4–256)
Pretransplant IgG titer (median, range)	8 (0–32)
Pretransplant IgM titer (median, range)	4 (1–32)
Plasma transfusion	23 (71.9%)
HLA MM (mean, SD)	3.09 (1.75)
Pretransplant ATG therapy	5 (15.6%)
Repeated kidney transplantation	0
Posttransplant infection, n (%)	9 (28.1%)
Lung	7 (21.9%)
Urinary tract	1 (3.1%)
BK virus	1 (3.1%)
Blood type mismatch (donor/recipient)	
A/O	11 (34.4%)
B/O	7 (21.9%)
A/B	2 (6.3%)
B/A	4 (12.5%)
AB/A	2 (6.3%)
AB/B	6 (18.8%)
Genotype match (donor/recipient)	
Secretor/secretor	18 (56.3%)
Secretor/non-secretor	9 (28.1%)
Non-secretor/secretor	5 (15.6%)
Non-secretor/non-secretor	0
Phenotype match (donor/recipient)	
Positive/positive	17 (53.1%)
Positive/weak	7 (21.9%)
Positive/negative	2 (6.3%)
Weak/positive	4 (12.5%)
Negative/positive	2 (6.3%)

IgG/IgM titers were transformed to log base 2.

PRA, panel reactive antibody; ATG, anti-thymocyte globulin; BMI, body mass index; SD, standard error.

The correlations between the fluctuation of anti-A/B antibody titers and donor or recipient secretor status are shown in **Table 3** and **Supplementary Material 1**, respectively. Overall, seven donors underwent IgG antibody titer elevation and 13 donors underwent IgM antibody titer elevation after transplantation, whereas IgG and IgM antibody titer reductions after transplantation were observed in 23 and 22 donors, respectively. However, there was no significant difference in the frequencies of titer elevation and reduction. A significant difference in the time required for one log2 IgM titer reduction was observed in recipients between the two genotype donor groups (from secreting genotype donors *vs*. from non-secreting genotype donors, P = 0.023). The number of days required for one log2 IgM titer reduction between the two phenotype donor groups (from positive secreting phenotype donors *vs*. from weak or negative secreting phenotype donors) presented the same trend but were not statistically different (p=0.053). However, the days required for one log2 elevation in IgG and IgM or reduction in IgG were not significantly different between the cohorts grouped by the four methods (**Table 3**).

**Table 3:** Anti-A/B antibody titers and renal function according to donors’ secretor status.

	N of recipients							
	Overall (n=32)	Genotypes			Phenotypes			
		From secretor donors (n=27)	From non-secretor donors (n=5)	P value	From secretor donors (n=26)	From weak or negative secretor donors (n=6)		P value
Anti A/B titer variation after transplantation								
Titer elevation, n (%)								
IgG	7 (21.9%)	5 (18.5%)	2 (40%)	0.296	5 (19.2%)		2 (33.3%)	0.590
IgM	13 (40.6%)	10 (37.0%)	3 (60%)	0.375	10 (38.5%)		3 (33.3%)	0.666
Median days to one log2 titer elevation								
IgG	7	7	10.5	0.554	7		10.5	0.554
IgM	7	7	7	1.000	7		7	1.00
Titer reduction, n (%)								
IgG	23 (71.9%)	20 (74.1%)	3 (60%)	0.604	19 (73.1%)		4 (66.7%)	1.000
IgM	22 (68.8%)	19 (70.4%)	3 (60%)	0.637	18 (69.2)		4 (66.7%)	1.000
Median days to one log2 titer reduction								
IgG	7	7	5	0.607	7		4.5	0.934
IgM	3	3	2	**0.023**	3		2	0.053
Scr reduction after transplantation								
Minimum level compared to initial Scr level (%)	11.6 ± 6.3	11.3 ± 5.1	13.2 ± 11.5	0.735	11.5 ± 5.2		12.3 ± 10.6	0.858
Absolute values of maximal Scr reduction	857 ± 302	832 ± 283	957 ± 412	0.545	831 ± 288		940 ± 370	0.524
Median days to minimum Scr level	7	7	7	0.096	7		5	**0.024**

Scr, serum creatinine.

The bold values indicate that the P value is less than 0.05.

**Figure 1:**
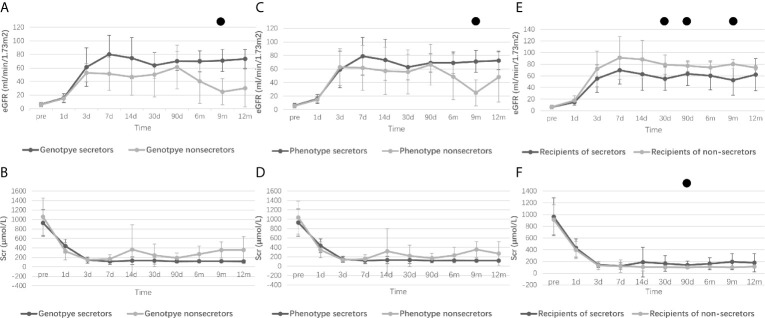
Perioperative renal function of recipients according to different grouping methods. Research data are presented as mean and standard deviation. Perioperative eGFR levels of recipients refer to donor genotypes [**(A)**, from genotype secretor donors *vs*. genotype non-secretor donors], donor phenotype [**(C)**, from phenotype secretor donors *vs*. phenotype weak- or non-secretor donors] and recipient secretor status [**(E)**, secretor recipients *vs*. non-secretor recipients]. Perioperative Scr levels of recipients refer to donor genotypes [**(B)**, from genotype secretor donors *vs*. genotype non-secretor donors], donor phenotype [**(D)**, from phenotype secretor donors *vs*. phenotype weak- or non-secretor donors] and recipient secretor status [**(F)**, secretor recipients *vs*. non-secretor recipients]. eGFR, Estimated glomerular filtration rate; Scr, Serum creatinine. ●: Difference is statistically significant between groups, and detailed P values are shown in **Supplementary Material 2**.

**Figure 1** displays the changes in eGFR and Scr after kidney transplantation, and ““**Supplementary Material 2** presents the corresponding P values of eGFR or Scr between groups according to different grouping methods. There was no statistical difference in pretransplant eGFR and Scr among the various grouping methods. However, recipients with weak or non-secretor status tended to have better eGFR at day 30 (P=0.008), day 90 (P=0.005), and month 9 (P=0.008) compared to those with secretor status. A lower Scr was also observed at day 30 (P=0.047) and day 90 (P=0.010) in the recipients with weak or non-secretors. Interestingly, recipients with non-secretors tended to spend more time (P=0.053) to reach a lower minimum Scr level (P=0.080), but the results were not statistically different (**Supplementary Material 1**). Moreover, recipients from phenotype secretors underwent shorter days to reach the minimum level of Scr compared to recipients from phenotype weak- or non-secretors (5 days vs. 7 days, P=0.024), as displayed in **Table 3**.

**Figure 2:**
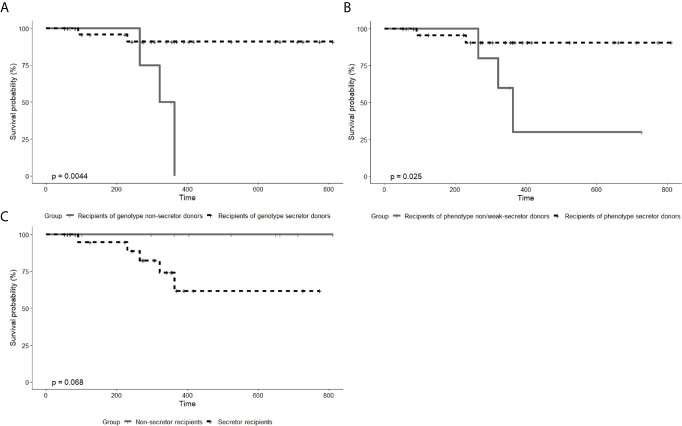
Kaplan–Meier curve of graft rejection between recipients from genotype secretor donors and from genotype non-secretor donors **(A)**, between recipients from phenotype secretor donors and from phenotype weak- or non-secretor donors **(B)**, and between secretor recipients and non-secretor recipients **(C)**. P values were calculated by log-rank tests. In both A and B, there are two patients diagnosed with graft rejection in the group of secretor donors and three patients in the group of non-secretor donors. Owing to the different number of patients by different grouping methods, the P values in **(A, B)** differ. In **(C)**, all the five patients clinically diagnosed with graft rejection are from the group of secretor recipients.

Biopsies were performed in 5 recipients who were clinically diagnosed with graft rejection, and then 3 recipients were biopsy-proved. Among those 5 recipients who developed rejection, only 1 recipient was with positive pretransplant PRA. Antibody-mediated rejection (ABMR) and T-cell-mediated rejection (TCMR) were observed in 2 and 1 recipients, respectively. The remaining 2 patients had typical clinical symptoms and remarkable Scr increase but the biopsy results showed no rejection. They were still regarded as recipients clinically diagnosed with graft rejection, owing to the typical clinical symptoms which cannot be explained by other reasons and the fact that they were treated for rejection before the biopsy. **Table 4** displays the number of recipients with graft rejection according to different grouping methods, and the differences between recipients of secretor donors and recipients of non-secretor donors were statistically significant (P=0.018 and P=0.034, Chi-square test). **Figure 2** shows the survival curves of graft rejection according to different secretor status of donors and recipients (survival curves of three biopsy-proven graft rejection are presented in **Supplementary 3**). Recipients of genotype and phenotype secretor donors have a statistical tendency to undergo better rejection-free survival. However, the follow-up time of the 2 recipients who did not have rejection from non-secretor genotype donors is relatively short, and 0% survival beyond 400 days is demonstrated on the Kaplan-Meier graph of **Figure 2A**.

**Table 4:** The number of recipients with graft rejection according to different grouping methods.

Grouping methods	Rejection, n (%)	Rejection-free, n (%)	P value
Donor genotypes			**0.018**
Secretor	2 (7.4%)	25 (92.6%)	
Non-secretor	3 (60%)	2 (40%)	
Donor phenotypes			**0.034**
Positive	2 (7.7%)	24 (92.3%)	
Weak or negative	3 (50%)	3 (50%)	
Recipient genotypes			0.288
Secretor	5 (21.7%)	18 (78.3%)	
Non-secretor	0 (0%)	9 (100%)	
Recipient phenotypes			0.288
Positive	5 (21.7%)	18 (78.3%)	
Weak or negative	0 (0%)	9 (100%)	

P values was calculated by log-rank tests.

The bold values indicate that the P value is less than 0.05.

## Discussion

**Figure 3:**
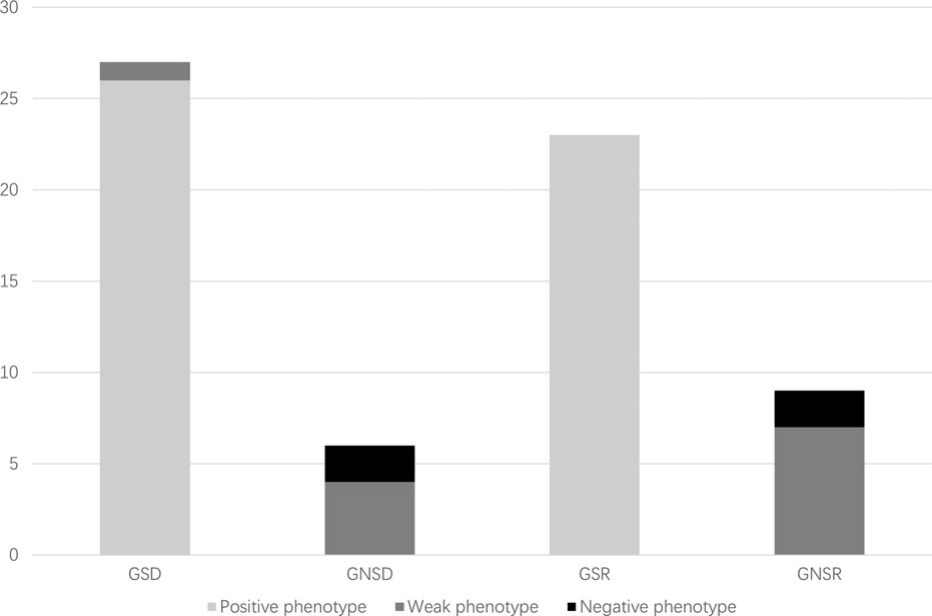
Distribution of secretor genotypes and phenotypes of study population. GSD, genotype secretor donors; GNSD, genotype non-secretor donors; GSR, genotype secretor recipients; GNSR, genotype non-secretor recipients.

The ABO blood group barrier was successfully broken down by proper desensitization protocols before kidney transplantation. Previous studies from several centers have reported comparable overall and graft survival rates between ABOi and ABOc transplantation (21–23). However, little attention has been paid to the influence of secretor status, and previous results are still controversial. In this study, we investigated the impact of donor and recipient secretor status on the outcomes of kidney transplantation. Post-transplant AMR is caused not only by ABO antibodies but also by HLA Class I and II antibodies. To lessen the impact of HLA antibodies, recipients with DSA were excluded from the study. Interestingly, the genotype and phenotype of the secretors were not fully consistent (**Figure 3**). The weak-secretors phenotype was found in both genotype secretors and non-secretors, which is common in South Asian human populations (24). This is a result of a weakly mutated form of the secretor transferase.

The grafts from secretor status donors are capable of secreting soluble A/B blood group antigens into the blood of recipients. Therefore, it is hypothesized that the soluble A/B blood group antigens may bind to the anti-A/B antibodies of recipients and reduce post-transplant titers, preventing the incidence of AMR. In addition to RBCs (red blood cells), ABO blood group antigens are also distributed on lymphocytes, platelets, most of the epithelial and endothelial cells, including the vascular endothelium, distal convoluted tubules and collecting ducts of the donor kidney (25). Posttransplant anti-A/B titers, which reflect the binding ability with antigens on the graft kidney, have been considered to play important roles in the incidence of AMR (26).

Most recipients underwent one or more titer reductions, implying good progress after transplantation. The reduction in titers may be due to the dilution caused by the expansion of the blood volume after surgery, the neutralization of the secreted antigen, or the binding and adsorption of the graft (27). Titer elevation was also found in some recipients, which was correlated with increased rates of graft loss (28). However, posttransplant titer elevation or reduction was not significantly related to the secretor status of donors, which aligned with the results of previous studies. Furthermore, Kim et al. found that posttransplant anti-A/B antibody titers were also not influenced by the secretor status of the donor; however, IgM titers showed a rapid decline in recipients from donors with the non-secretor (17). We observed a similar trend of rapid IgM reduction in our study. This could be explained by the lack of absorption of soluble antigen from the non-secreting graft, and subsequently the binding of antibody and antigen in the allograft was enhanced. However, this finding may be affected by the frequency of IgM titer checks in the posttransplant period and requires another study with more cases and more frequent titer evaluations. Additionally, the reduction in IgG was not significantly different between the secreting and non-secreting donor groups. This may be due to the predominant type IgM in anti-A/B antibodies, and therefore, IgM may be more affected by soluble ABH absorption (29).

In this study, we found that recipients of secretor donors tended to experience better renal function and lower incidence of graft rejection, but the results regarding improvement of renal function between the two groups were not statistically significant. Pertaining to this point, the results of previous studies are contradictory. Drexler et al. believed that patients who received kidney transplantation from a secretor donor underwent significant improvement in early renal function after transplantation, with a significant impact on humoral rejection (16). However, Kim et al. found that soluble ABH antigens produced by grafts from secretor donors did not affect renal function and graft rejection in recipients (17). In our opinion, even though posttransplant anti-A/B titers are not influenced by the secretor status of the donor, soluble antigens secreted by the graft of the secretor donor may continuously bind circulating blood group antibodies, having a protective impact on renal function and preventing graft rejection.

In addition to the secretor status of donors, we investigated the correlation between recipient secretor status and posttransplant prognosis. To the best of our knowledge, this is the first study to assess the impact of recipient secretor status on the outcomes of ABOi kidney transplantation. Posttransplant anti-A/B titers were not affected by recipient secretor status, similar to donor secretor status. However, we discovered that renal function was considerably influenced by the secretor status of recipients. These weak- or non-secretor recipients experienced better renal function and a trend towards a lower rate of graft rejection. We then hypothesized that the secretor status of recipients might somehow result in a change in anti-A/B antibodies and influence graft function. Overall, the exact mechanisms by which non-secretor phenotype recipients experience better renal function remain unknown and require further research.

The main limitations of our study include its retrospective design and relatively short follow-up time. In addition, owing to the relatively recent development of ABOi kidney transplantation technology, our study was limited by the number of samples. Technical issues of titer measurement may also influence the results. To minimize this bias, we chose a relatively precise gel card technique rather than the conventional tube method. The observations and subsequent inferences can be influenced by all these factors; therefore, more data of patients with longer follow-up periods should be collected and would be of great value.

In conclusion, the genotype and phenotype of the determined secretor status do not completely correspond with each other. Anti-A/B titers after ABOi kidney transplantation were not influenced by the secretor status of the donors and recipients. However, the recipient ABH-secretor status may have an influence on early posttransplant renal function and the donor ABH-secretor status might affect the incidence of graft rejection.

## Data Availability Statement

The original contributions presented in the study are included in the article/**Supplementary Material**. Further inquiries can be directed to the corresponding authors.

## Ethics Statement

The studies involving human participants were reviewed and approved by the West China Hospital Ethics Committee (no. 2019SHEN418). The patients/participants provided their written informed consent to participate in this study.

## Author Contributions

FZ, SY, and XW contributed to the central idea and coordinated the writing of the manuscript. XW and TL read, discussed, and revised the manuscript. YF, TS, ZH, JL, JW, and YY helped collect the samples and data. All authors contributed to the article and approved the submitted version.

## Funding

This work was supported by grants from the National Natural Science Foundation of China [grant number 81870513], Sichuan Science and Technology Program [grant number 2019YJ0133], Chengdu Science and Technology Program [grant number 2019-YF05-00084-SN], and 1.3.5 Project for Disciplines of Excellence-Clinical Research Incubation Project, West China Hospital, Sichuan University [grant numbers 2018HXFH049, ZYJC18004, ZY2016104, 2021HXFH007]. The funders had no role in the study design, data collection or analysis, preparation of the manuscript, or the decision to publish.

## Conflict of Interest

The authors declare that the research was conducted in the absence of any commercial or financial relationships that could be construed as a potential conflict of interest.

## Supplementary Material

Supplementary Material 3 Kaplan–Meier curve of biopsy-proven graft rejection between recipients from genotype secretor donors and from genotype non-secretor donors **(A)**, between recipients from phenotype secretor donors and from phenotype weak- or non-secretor donors **(B)**, and between secretor recipients and non-secretor recipients **(C)**. P values were calculated using log-rank tests.
