# Supplementary figures and images for: Effect of Donor and Recipient ABH-Secretor Status on ABO-Incompatible Living Donor Kidney Transplantation
Source: Front Immunol. 2021 Jun 14;12:671185. doi: 10.3389/fimmu.2021.671185 (PMC8236826; doi:10.3389/fimmu.2021.671185)

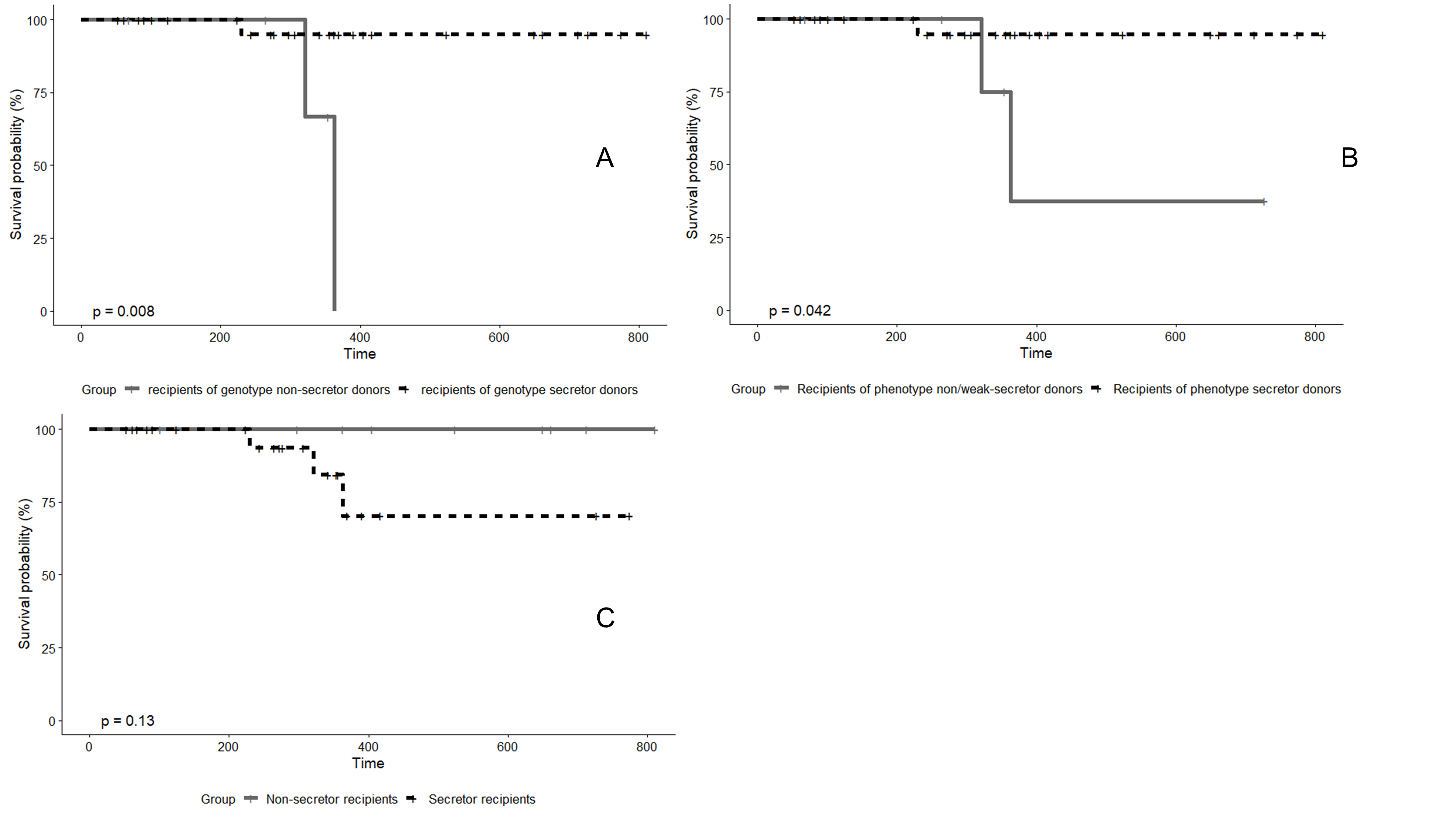

Supplement: Supplementary file 1 [file Image_1.tif]
