# Supplementary material for: Effect of Donor and Recipient ABH-Secretor Status on ABO-Incompatible Living Donor Kidney Transplantation
Source: Front Immunol. 2021 Jun 14;12:671185. doi: 10.3389/fimmu.2021.671185 (PMC8236826; doi:10.3389/fimmu.2021.671185)
Supplement: Supplementary file 2 [file Table_1.docx]

Supplementary material 1: Anti-A/B antibody titers and renal function according to recipients’ secretor status

|  |  |  | N of recipients | | | | | | | |
| --- | --- | --- | --- | --- | --- | --- | --- | --- | --- | --- |
|  |  |  | Overall (n=32) | Genotypes | | |  | Phenotypes | | |
|  |  |  |  | For secretor recipients (n=23) | For non-secretor recipients (n=9) | P value |  | For secretor recipients (n=23) | For weak or negative secretor recipients (n=9) | P value |
| Anti A/B titer variation after transplantation | | |  |  |  |  |  |  |  |  |
|  | Titer elevation, n (%) | |  |  |  |  |  |  |  |  |
|  |  | IgG | 7 (21.9%) | 6 (26.1%) | 1 (11.1%) | 0.640 |  | 6 (26.1%) | 1 (11.1%) | 0.640 |
|  |  | IgM | 13 (40.6%) | 9 (39.1%) | 4 (44.4%) | 1.000 |  | 9 (39.1%) | 4 (44.4%) | 1.000 |
|  | Median days to one log2 titer elevation | |  |  |  |  |  |  |  |  |
|  |  | IgG | 7 | 8 | 5 | 0.203 |  | 8 | 5 | 0.203 |
|  |  | IgM | 7 | 7 | 23 | 0.579 |  | 7 | 23 | 0.579 |
|  | Titer reduction, n (%) | |  |  |  |  |  |  |  |  |
|  |  | IgG | 23 (71.9%) | 16 (69.6%) | 7 (77.8%) | 1.000 |  | 16 (69.6%) | 7 (77.8%) | 1.000 |
|  |  | IgM | 22 (68.8%) | 16 (69.6%) | 6 (66.7%) | 1.000 |  | 16 (69.6%) | 6 (66.7%) | 1.000 |
|  | Median days to one log2 titer reduction | |  |  |  |  |  |  |  |  |
|  |  | IgG | 7 | 7 | 5 | 0.891 |  | 7 | 5 | 0.891 |
|  |  | IgM | 3 | 3 | 3 | 0.349 |  | 3 | 3 | 0.349 |
| Scr reduction after transplantation | | |  |  |  |  |  |  |  |  |
|  | Minimum level compared to initial Scr level (%) | | 11.6 ± 6.3 | 12.6 ± 7.1 | 9.2 ± 2.4 | 0.053 |  | 12.6 ± 7.1 | 9.2 ± 2.4 | 0.053 |
|  | Absolute values of maximal Scr reduction | | 857 ± 302 | 859 ± 327 | 834 ± 240 | 0.815 |  | 859 ± 327 | 859 ± 327 | 0.815 |
|  | Median days to minimum Scr level | | 7 | 7 | 14 | 0.080 |  | 7 | 14 | 0.080 |

Scr: serum creatinine.
