# Supplementary material for: Effect of Donor and Recipient ABH-Secretor Status on ABO-Incompatible Living Donor Kidney Transplantation
Source: Front Immunol. 2021 Jun 14;12:671185. doi: 10.3389/fimmu.2021.671185 (PMC8236826; doi:10.3389/fimmu.2021.671185)
Supplement: Supplementary Material 3 — Kaplan–Meier curve of biopsy-proven graft rejection between recipients from genotype secretor donors and from genotype non-secretor donors (A), between recipients from phenotype secretor donors and from phenotype weak- or non-secretor donors (B), and between secretor recipients and non-secretor recipients (C). P values were calculated using log-rank tests. [file Table_2.docx]

Supplementary material 2: P values of perioperative GFR or Scr between groups according to different grouping methods

|  | eGFR | | |  | Scr | | |
| --- | --- | --- | --- | --- | --- | --- | --- |
|  | Donor genotypes | Donor phenotypes | Recipient |  | Donor genotypes | Donor phenotypes | Recipient |
| pre | 0.788 | 0.703 | 0.913 |  | 0.517 | 0.518 | 0.648 |
| 1d | 0.798 | 0.645 | 0.304 |  | 0.264 | 0.283 | 0.591 |
| 3d | 0.321 | 0.787 | 0.163 |  | 0.774 | 0.607 | 0.555 |
| 7d | 0.054* | 0.271 | 0.136 |  | 0.265 | 0.397 | 0.922 |
| 14d | 0.084* | 0.329 | 0.058* |  | 0.367 | 0.386 | 0.171 |
| 30d | 0.412 | 0.623 | **0.008** |  | 0.346 | 0.377 | **0.047** |
| 90d | 0.624 | 0.859 | **0.005** |  | 0.212 | 0.254 | **0.010** |
| 6m | 0.168 | 0.250 | 0.060 |  | 0.176 | 0.199 | 0.061* |
| 9m | **0.036** | **0.036** | **0.008** |  | 0.139 | 0.139 | 0.059* |
| 12m | 0.262 | 0.368 | 0.271 |  | 0.446 | 0.407 | 0.262 |

GFR: glomerular filtration rate; Scr: serum creatinine. P values are calculated by student t tests.
